# Genome-Wide Mapping of Binding Sites Reveals Multiple Biological Functions of the Transcription Factor Cst6p in *Saccharomyces cerevisiae*

**DOI:** 10.1128/mBio.00559-16

**Published:** 2016-05-03

**Authors:** Guodong Liu, David Bergenholm, Jens Nielsen

**Affiliations:** a Department of Biology and Biological Engineering, Novo Nordisk Foundation Center for Biosustainability, Chalmers University of Technology, Gothenburg, Sweden; b Novo Nordisk Foundation Center for Biosustainability, Technical University of Denmark, Hørsholm, Denmark

## Abstract

In the model eukaryote *Saccharomyces cerevisiae*, the transcription factor Cst6p has been reported to play important roles in several biological processes. However, the genome-wide targets of Cst6p and its physiological functions remain unknown. Here, we mapped the genome-wide binding sites of Cst6p at high resolution. Cst6p binds to the promoter regions of 59 genes with various biological functions when cells are grown on ethanol but hardly binds to the promoter at any gene when cells are grown on glucose. The retarded growth of the *CST6* deletion mutant on ethanol is attributed to the markedly decreased expression of *NCE103*, encoding a carbonic anhydrase, which is a direct target of Cst6p. The target genes of Cst6p have a large overlap with those of stress-responsive transcription factors, such as Sko1p and Skn7p. In addition, a *CST6* deletion mutant growing on ethanol shows hypersensitivity to oxidative stress and ethanol stress, assigning Cst6p as a new member of the stress-responsive transcriptional regulatory network. These results show that mapping of genome-wide binding sites can provide new insights into the function of transcription factors and highlight the highly connected and condition-dependent nature of the transcriptional regulatory network in *S. cerevisiae*.

## IMPORTANCE

Transcription factors regulate the activity of various biological processes through binding to specific DNA sequences. Therefore, the determination of binding positions is important for the understanding of the regulatory effects of transcription factors. In the model eukaryote *Saccharomyces cerevisiae*, the transcription factor Cst6p has been reported to regulate several biological processes, while its genome-wide targets remain unknown. Here, we mapped the genome-wide binding sites of Cst6p at high resolution. We show that the binding of Cst6p to its target promoters is condition dependent and explain the mechanism for the retarded growth of the *CST6* deletion mutant on ethanol. Furthermore, we demonstrate that Cst6p is a new member of a stress-responsive transcriptional regulatory network. These results provide deeper understanding of the function of the dynamic transcriptional regulatory network in *S. cerevisiae*.

## INTRODUCTION

Transcription factors (TFs) bind to specific DNA sequences to affect the transcription status of nearby genes and, thus, regulate the activities of related biological processes. In the model eukaryote *Saccharomyces cerevisiae*, about 209 sequence-specific TFs have been characterized or predicted (1), and their roles in regulating metabolism, cell cycle, development, and stress responses were reported. The determination of binding positions is critical for the understanding of the regulatory effects of TFs (2). By using genome-wide methods for mapping of TF binding sites, e.g., chromatin immunoprecipitation with microarray technology (ChIP-chip) analysis (3), it is possible to get a global view of all genes controlled by a given TF (4). The binding of many TFs is, however, strongly dependent on the environmental conditions, and mapping of binding sites should therefore best be performed under various physiological conditions.

In *S. cerevisiae*, Cst6p is an ATF/CREB family TF with a basic leucine zipper (bZIP) domain. All three members in this family, Sko1p, Aca1p, and Cst6p (alias Aca2p), bind to the 5′ TGACGTCA 3′ sequence *in vitro* (5). The function of Sko1p in osmotic and oxidative stress responses and its genome-wide regulatory network have been well documented (6–8). However, the regulatory roles of Aca1p and Cst6p, which are paralogs derived from whole-genome duplication, are less clear. Cst6p binds to DNA as a homodimer or a heterodimer together with Aca1p and shows transcriptional activating ability (5). Phenotypic analyses and genome-wide screening have revealed that Cst6p has functions in several different cellular processes. Deletion of *CST6* leads to poor or no growth on respiratory carbon sources like glycerol, ethanol, and raffinose (5). Phosphorylation of Cst6p is induced by oleate, and a constitutively nonphosphorylated mutant of Cst6p results in increased expression of β-oxidation genes (9). In addition, a *CST6* deletion mutant shows poor ability to maintain 2µm plasmids (10), while its overexpression results in chromosome instability (11). Currently, the only well-studied direct target of Cst6p is *NCE103* (12), which encodes a carbonic anhydrase converting CO_2_ to HCO_3_^−^, the latter serving as a substrate of several carboxylation reactions. Under low-CO_2_ conditions, Cst6p binds to the promoter of *NCE103* and activates its expression. In some other fungal species, the orthologs of Cst6p have pleiotropic functions. Thus, in *Candida albicans*, Rca1p also directly activates carbonic anhydrase gene expression (12), with other phenotypes being altered hyphal formation, membrane ergosterol content, antifungal responses, and chemical resistance (12, 13). In *Candida glabrata*, Cst6p negatively regulates the expression of the main adhesin gene *EPA6* and, thereby, biofilm formation (14).

Despite extensive phenotypic studies on *CST6* mutants, the genome-wide *in vivo* targets of Cst6p still remain unknown. The ChIP-chip study of 203 TFs in *S. cerevisiae* (4) included Cst6p, but the target list identified seems questionable, because the known target *NCE103* was not found and no consensus binding motif could be enriched from the target sequences. We therefore mapped the binding sites of Cst6p during growth on both glucose and ethanol, using ChIP with lambda exonuclease digestion followed by sequencing (ChIP-exo), which allows for identifying the location of DNA-binding proteins at high resolution (15). Following this, we measured the regulatory effect of Cst6p on its target genes, and the biological functions of these regulatory effects were investigated. Our results provide deeper understanding of the function of Cst6p and how it integrates with the transcriptional regulatory network in *S. cerevisiae*.

## RESULTS

### Identification of Cst6p binding sites by ChIP-exo.

**FIG 1:**
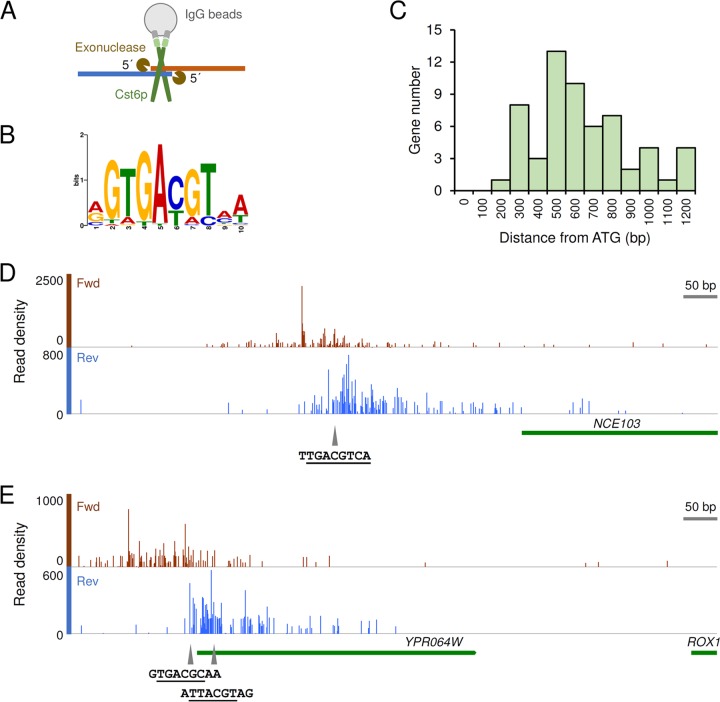
The identification of Cst6p binding sites by ChIP-exo. (A) Schematic representation of the ChIP-exo experiment. Cst6p with C-terminal tag is shown in the form of a homodimer. (B) Binding site motif enriched from the bound region sequences (*E* value = 8.9 × 10^−10^). (C) Distribution of the distances of binding events from the start codons of putative target genes. (D) Binding of Cst6p on the *NCE103* promoter. (E) Binding of Cst6p on the *ROX1* promoter. *YPR064W* is a dubious gene upstream from *ROX1*. Data shown in panels B to E are based on the binding events that occurred when cells were grown on ethanol. In panels D and E, the coverage graph showing the 5′ ends of reads was created from the aligned BAM file using “Depth Graph (Start)” in IGB (46). The putative binding sites of Cst6p are marked by triangles. The core binding sequences of the ATF/CREB family TFs (consensus sequence, 5′ TGACGT 3′) are underlined.

A *cst6*Δ strain was reported to have a more severe growth defect on respiratory carbon sources (including ethanol and galactose) than on glucose (5, 16), indicating a stronger role of this TF during growth on ethanol than during growth on glucose. We therefore aimed to identify the binding targets of Cst6p during growth on ethanol. To enable immunoprecipitation of Cst6p, we tagged it with CBP-ProtA *in situ*. The tag does not seem to affect the function of Cst6p during growth on ethanol, as growth was unaffected (see Fig. S1 in the supplemental material). Using ChIP-exo (Fig. 1A), we identified 40 binding sites distributed on 14 chromosomes when the cells were grown in synthetic medium with ethanol as the carbon source in batch cultivation (see Table S1 in the supplemental material). A binding site motif of 5′ GTGACGT 3′ was enriched from the bound region sequences (discovered in 32 of the 40 regions) (Fig. 1B). Compared with the motif determined by an *in vitro* protein binding microarray study (5′ TGACGT 3′) (17), an additional conserved guanine nucleotide was found, indicating different preferences of DNA binding between Cst6p and Sko1p (5′ ATGACGT 3′) (18). For growth on glucose, only 6 binding events with low signal-to-noise ratios were detected, of which 4 were also found during growth on ethanol (see Table S1), indicating that Cst6p hardly binds to its targets during growth on glucose. In the following analysis, we therefore only focus on the targets identified during growth on ethanol.

Of the 40 binding sites identified for growth on ethanol, 38 were located in putative promoter regions of genes, suggesting a general regulatory function of these bindings. Due to the presence of divergent promoters, a total of 59 protein-coding genes were identified as putative targets for Cst6p regulation (see Table S2 in the supplemental material). When this gene list was compared with the 106 Cst6p targets (*P* value ≤0.01) identified by ChIP-chip for growth in yeast extract-peptone-dextrose (YPD) medium (4), no overlap was observed. We suppose that the Cst6p tagging or the ChIP process did not work properly in the ChIP-chip study, though there is also the possibility that the targets of Cst6p on YPD are totally different from those on synthetic medium which we used. We also compared our ChIP-exo gene list with a list of orthologue genes in *C. albicans* found to be bound by Rca1p, the Cst6p orthologue in this yeast, during growth in YPD medium by using ChIP-chip with tiling microarray (12). Only *NCE103* was shared by the two datasets. Whether this large difference is due to the condition-dependent characteristic of Cst6p binding or to the rapid evolution of regulatory networks in fungi (19) is worth exploration.

The exonuclease digestion in ChIP-exo allowed high-resolution mapping of Cst6p locations. Overall, the binding sites have an average distance of 591 bp from the start codons (Fig. 1C). The previously suggested binding site on the *NCE103* promoter (12) was clearly found among the sequencing read peaks on both the forward and reverse strands (Fig. 1D). Multiple peaks were observed on the promoter region of *ROX1*, which encodes a transcriptional repressor of hypoxic genes, probably due to the presence of two binding sites of Cst6p located close to each other (Fig. 1E) or, perhaps, to the occupancy of other proteins at this area (20). Using the results of ChIP-exo, it is possible to validate or exclude computationally predicted binding sites on gene promoters. For example, the Cst6p binding motif 5′ GTGACGT 3′ located 982 bp upstream from the start codon of *ACC1* was identified as a real binding site, while no binding was found at another putative binding site (5′ TTGACGT 3′, 807 bp upstream from *ACC1*).

**FIG 2:**
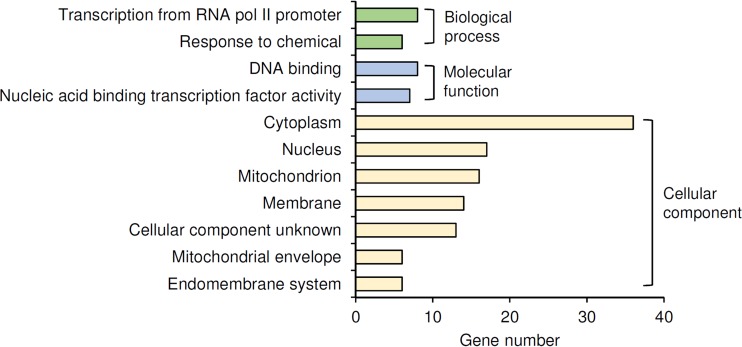
GO Slim annotation of the targets of Cst6p on ethanol. Only terms with more than six target genes (10% of total targets) are shown. All the GO terms annotated to the genes are listed in Table S3 in the supplemental material.

Gene ontology analysis of the 59 target genes (Fig. 2; see also Table S3 in the supplemental material) revealed two notable features: (i) 10 of the targets (*CBF2*, *GAT2*, *HAP4*, *PHD1*, *RMI1*, *ROX1*, *SIR4*, *SOK2*, *YAP1*, and *YAP6*) encode DNA-binding or transcriptional regulatory proteins, and (ii) 16 of the targets encode mitochondrial proteins, including six located in the mitochondrial envelope. In terms of cellular processes, the target genes are categorized into cellular respiration, gluconeogenesis, stress response, and pseudohyphal growth. Interestingly, in addition to *NCE103*, Cst6p binds to the promoters of *ACC1* and *PYC1*, whose protein products are major HCO_3_^−^-consuming enzymes in *S. cerevisiae*.

### Cst6p functionally regulates the expression of its targets.

**FIG 3:**
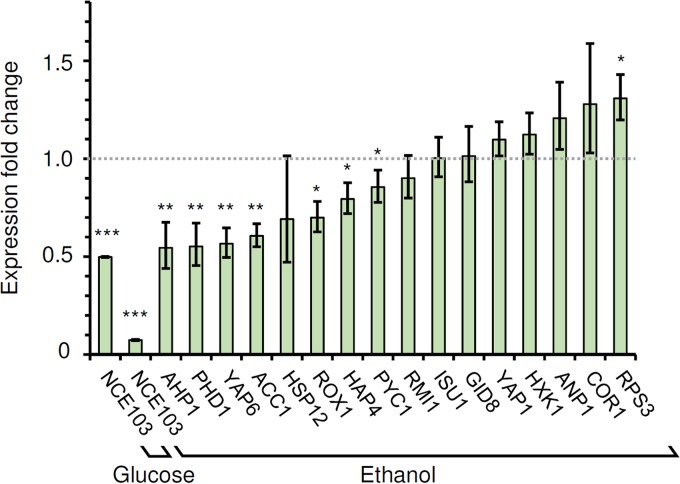
Fold changes in expression levels of Cst6p target genes in the *cst6*Δ strain relative to their expression in the wild type. The data represent the average results and standard deviations from biological triplicates. *, *P* ≤ 0.05; **, *P* ≤ 0.01; ***, *P* ≤ 0.001. A fold change of one (no change) is indicated by the dotted line.

To investigate the regulatory effects of the Cst6p binding events identified, the expression levels of 17 target genes in the *cst6*Δ strain were compared with their expression levels in the wild type. The *CST6* deletion mutant in the CEN.PK genetic background is viable on ethanol (see below), allowing the measurement of gene expression under this growth condition where we found Cst6p to be most active. As expected, the expression of *CST6* itself was not detected in the *cst6*Δ strain (data not shown). The expression of *NCE103* had a 92.6% decrease in the *cst6*Δ strain relative to its expression in the wild type, while a smaller decrease of 50.2% was observed during growth on glucose (Fig. 3). For the other target genes investigated, seven (*AHP1*, *PHD1*, *YAP6*, *ACC1*, *ROX1*, *HAP4*, and *PYC1*) had significantly decreased expression (approximate 15% to 45%) relative to their expression in the wild type for growth on ethanol. *RPS3* had a 30.9% increase in expression, while the other eight genes tested did not show significant changes in expression between the *cst6*Δ strain and the wild type. No relation could be found between the extent of the expression changes of the target genes and the binding strength (signal-to-noise ratio of ChIP-exo peaks) of Cst6p on their promoters.

### The function of Cst6p during growth on ethanol is dependent on *NCE103* expression.

**FIG 4:**
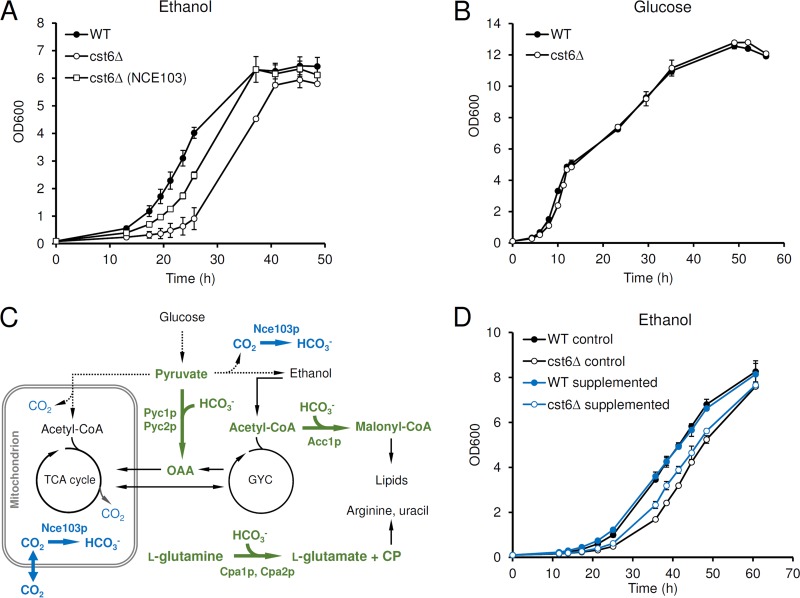
The function of Cst6p during growth on ethanol. (A) Growth on 1% (vol/vol) ethanol. (B) Growth on 2% (wt/vol) glucose. (C) Major pathways for CO_2_ and HCO_3_^−^ metabolism in *S. cerevisiae*. The reactions producing or consuming HCO_3_^−^ are shown by thick arrows. Pathways active on glucose are shown by dotted arrows. OAA, oxaloacetate; TCA cycle, tricarboxylic acid cycle; GYC, glyoxylate cycle; CoA, coenzyme A; CP, carbamoyl phosphate. (D) Growth on 1% (vol/vol) ethanol supplemented with or without fatty acids, uracil, and l-arginine. The growth with supplementation of ethanol-Tween 20 (see Materials and Methods) was used as a control. Supplementation increases the concentration of ethanol to 1.5% (vol/vol) and results in a higher final biomass concentration. The data represent the average results and standard deviations from biological duplications.

In a medium with 1% (vol/vol) ethanol as the sole carbon source, the *cst6*Δ strain had a longer lag phase, whereas the specific growth rate was only slightly lower later in the cultivation (Fig. 4A). This growth defect is less severe than that reported earlier. Thus, Garcia-Gimeno and Struhl observed no growth on ethanol (5), probably due to differences in strain backgrounds and in ethanol concentrations (3% ethanol was used in their study). In a glucose medium, the growth of the *cst6*Δ strain was similar to that of the wild type and respiratory growth after the diauxic shift was not affected (Fig. 4B).

Since the expression of *NCE103* was decreased to a greater extent than the expression of the other target genes with growth on ethanol (Fig. 3), we evaluated whether the low expression of this gene was contributing to the slower growth of the *cst6*Δ strain. Constitutive expression of *NCE103* using the *TEF1* promoter in the *cst6*Δ strain partially restored the growth on ethanol at the early stage (Fig. 4A), and this implies that the carbonic anhydrase activity or the subsequent HCO_3_^−^ concentration in the *cst6*Δ strain is the limiting factor for the initial growth of the *cst6*Δ strain on ethanol. The physiological role of Nce103p has been well documented: the *NCE103* deletion mutant is inviable in atmospheric air but can be complemented by supplementing the medium with some nutrients whose biosynthesis needs HCO_3_^−^, such as fatty acids, l-aspartic acid (providing oxaloacetate), uracil, and l-arginine (Fig. 4C) (21). Since the supplementation of l-aspartic acid is not essential for the complementation of the *nce103*Δ strain grown on ethanol (21), we chose fatty acids, uracil, and l-arginine to test whether they could also improve the growth of the *cst6*Δ strain on ethanol. While the growth of the wild type was slightly affected by the supplementation of nutrients, the *cst6*Δ strain did show enhanced growth (Fig. 4D).

### Cst6p is a member of the stress-responsive transcriptional regulatory network.

**FIG 5:**
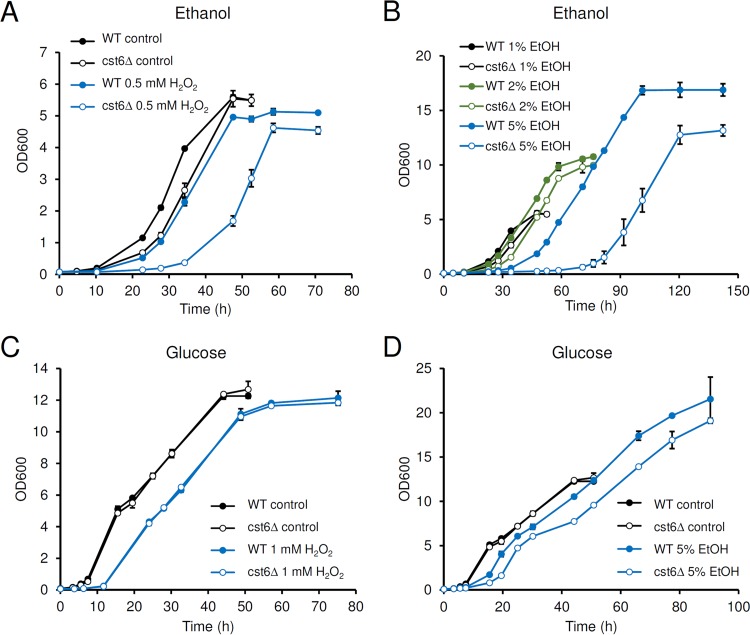
The function of Cst6p in stress resistance. (A) Growth on 1% (vol/vol) ethanol (EtOH) with or without supplementation of 0.5 mM H_2_O_2_. (B) Growth on different concentrations of ethanol. (C) Growth on 2% (wt/vol) glucose with or without supplementation of H_2_O_2_. (D) Growth on 2% (wt/vol) glucose with or without supplementation of ethanol. The data represent the average results and standard deviations from biological duplications.

Since Cst6p and Aca1p bind to a sequence similar to the sequence bound by the stress-responsive Sko1p *in vitro*, they were at first supposed to be involved in response to stress. However, phenotypic and gene expression analyses showed that Cst6p and Aca1p are not involved in the responses to various stresses (5). Another study reached a similar conclusion based on studies of osmotic stress but showed that the *cst6*Δ strain is more sensitive to oxidative stress than the wild type (22). Both these studies used YPD medium, and an effect of the brand of yeast extract and peptone on phenotype was noticed in the latter study. As our ChIP-exo results show that Cst6p binds to some stress-related genes during growth on ethanol, we wondered if Cst6p is involved in stress response under this condition. Growth test on ethanol showed that the *cst6*Δ strain is more sensitive than the wild type to H_2_O_2_ (Fig. 5A) and high concentrations of ethanol (Fig. 5B). On glucose, the *cst6*Δ strain showed sensitivity to H_2_O_2_ similar to that of the wild type (Fig. 5C). Hypersensitivity to ethanol stress was also observed for the *cst6*Δ strain grown on glucose but was less apparent than when it was grown on ethanol (Fig. 5D).

**FIG 6:**
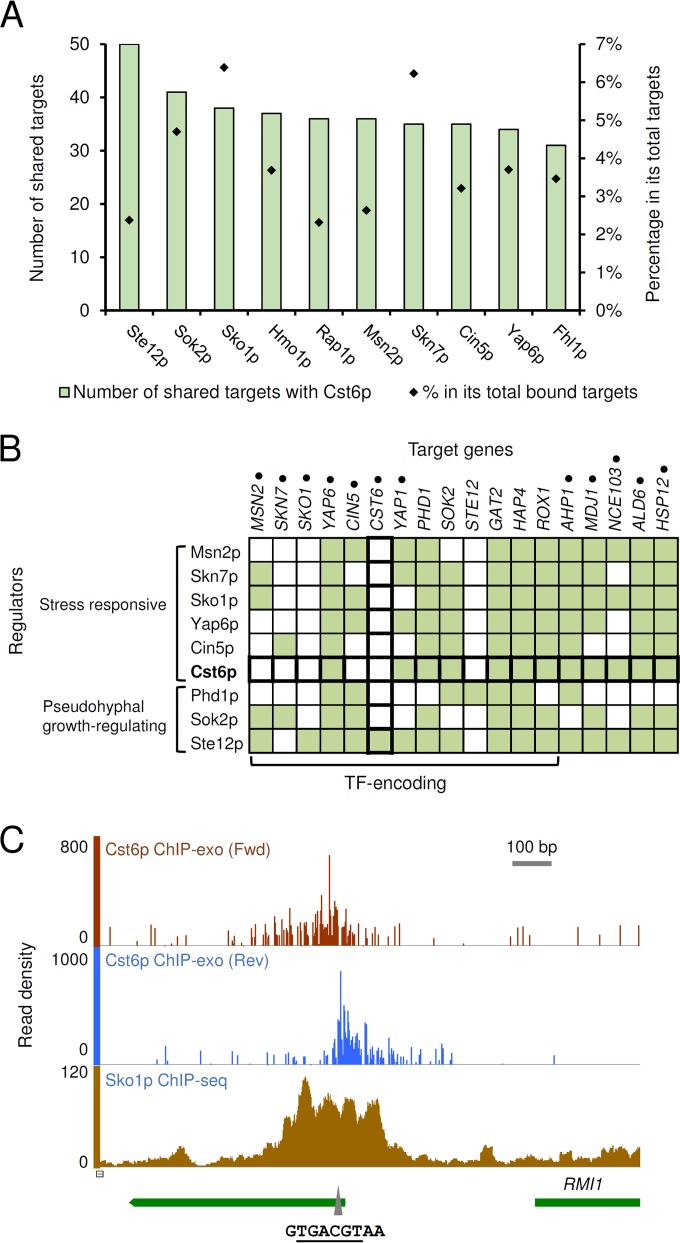
Cst6p as a member in the stress-response transcriptional regulatory network. (A) Top 10 TFs with the highest number of targets shared with Cst6p. All the coregulatory TFs and their shared targets with Cst6p are listed in Table S4 in the supplemental material. (B) Heat map showing coregulation of Cst6p targets. For regulators, 7 of the TFs in panel A and Phd1p are included. For target genes, the nine regulators themselves and Cst6p targets encoding TFs or stress-related proteins are included. Targets with known functions in stress response are marked by dots. Filled squares indicate TF-target binding relationships. (C) Binding of Cst6p (on ethanol) and Sko1 (under 0.4 M KCl stress) at the *SKS1*-*RMI1* divergent promoter region. The putative binding site of ATF/CREB family TFs (5′ TGACGT 3′) in this region is marked by the triangle.

This indicates that Cst6p is indeed a member of the stress-response regulatory network in *S. cerevisiae*. We therefore explored the relationship between Cst6p and other members of this extensively studied network (23, 24). As mentioned above, *YAP1* and *YAP6*, encoding stress-responsive TFs, are direct targets of Cst6p. Furthermore, overlap analysis of the Cst6p targets with the known bound targets of all the TFs in *S. cerevisiae* suggests that Cst6p coregulates gene expression with 106 other TFs (see Table S4 in the supplemental material). Notably, among the top 10 TFs with the highest number of shared targets with Cst6p (Fig. 6A), five (Sko1p, Msn2p, Skn7p, Cin5p, and Yap6p) are known as stress-responsive TFs and two (Ste12 and Sok2p) are involved in the regulation of pseudohyphal growth. The targets of Cst6p with extensive coregulation include the peroxiredoxin-encoding *AHP1*, heat shock protein-encoding *HSP12*, molecular chaperone-encoding *MDJ1*, and stress-responsive TF genes like *YAP6* (Fig. 6B).

Considering that Cst6p and Sko1p bind to the same sequence *in vitro*, we compared the binding sites for Cst6p that we identified in the work presented here with those identified by chromatin immunoprecipitation sequencing (ChIP-seq) for Sko1p (8). For most of the shared targets, the ChIP-exo peak of Cst6p was found inside the broader ChIP-seq peak region of Sko1p. Combined with the result of binding motif search, the two TFs appear to bind to the same site (Fig. 6C shows an example). On the other hand, some well-studied direct targets of Sko1p, such as *GRE2* (22), were not identified as direct targets of Cst6p.

## DISCUSSION

TFs in *S. cerevisiae* have been studied extensively for their functions and the underlying molecular mechanisms. However, knowledge of the specific functions of some TFs is still limited, and therefore, we performed a genome-wide binding-site mapping of Cst6p. Using this information, we revealed its role in regulating diverse cellular processes that can explain different phenotypes reported earlier and further studied here. For example, the binding of Cst6p to chromosome segregation-related genes *CBF2*, *GIP3*, and *RMI1* could be linked to the chromosome instability of a *CST6* overexpression mutant. Furthermore, we find that the regulatory function of Cst6p is highly condition dependent, as almost no binding event was identified in cells grown on glucose but several were identified in cells grown on ethanol.

From our analysis, the carbonic anhydrase gene *NCE103* was for the first time identified as a direct target of Cst6p in *S. cerevisiae*, although a putative Cst6p binding site on its promoter has been shown to be responsible for the regulation by CO_2_ (12). From quantitative analysis of gene expression, we showed that Cst6p contributes to most of the activation of *NCE103* expression for growth on ethanol (Fig. 3). This is consistent with the result in *C. albicans*, where Rca1p (ortholog of Cst6p) was identified as the only TF activating *NCE103* expression by screening a TF knockout mutant library (12). Furthermore, the Cst6p regulation of *NCE103* expression is the major determinant of the phenotype of a *CST6* deletion strain grown on ethanol (Fig. 4A). While *NCE103* is essential for HCO_3_^−^ supply and growth under atmospheric air, the phenotype of the *cst6*Δ strain is only obvious at the early stage of the growth on ethanol. When ethanol is used as the sole carbon source, the slow CO_2_ production by respiration (Fig. 4C) and the markedly decreased Nce103p level may not be able to provide enough HCO_3_^−^ for key biosynthetic reactions required for cell growth. The growth on glucose is almost not affected, probably due to the fact that *NCE103* expression is not significantly affected during growth on this carbon source (Fig. 3) or because the rapid CO_2_ production from fermentation ensures sufficient provision of HCO_3_^−^. In *C. albicans*, growth delay in the early stage was also observed for the *rca1*Δ mutant grown on glucose, especially when a synthetic medium was used (13). Taken together, the results suggest that Cst6p/Rca1p is important for the growth of yeast under conditions with low carbonate levels and that this is due to its activation of the conserved target *NCE103*. From a biotechnological point of view, the expression level of the carbonic anhydrase gene *NCE103* is a potential target for metabolic engineering to increase the production of related metabolites (Fig. 4C), and Cst6p could be used to design regulatory circuits that are responsive to CO_2_ levels.

The binding to stress-related genes and the results of stress resistance tests establish Cst6p as a stress-responsive TF. As seen by the results in Fig. 6B, the binding of several TFs to target genes associated with stress response makes the stress-responsive regulatory network in *S. cerevisiae* complex. Genetic analyses have given greater functional understanding of this network: the contributions of different TFs to the transcription of a specific gene are quite different, and the regulatory effect is critically dependent on the growth condition and type of stress (8, 22). From our results, Cst6p is positively involved in the expression of its stress-related targets during growth on ethanol, where oxidative stress might arise from respiration in the mitochondria (25). Except for *NCE103*, the target genes identified have moderate decreases in expression in the *cst6*Δ strain (Fig. 3), indicating the presence of other transcriptional activators or repressors controlling the expression of these genes. The number of binding sites, binding strengths, and regulatory effects of Cst6p may increase if the cells are exposed to a higher level stress (e.g., the addition of H_2_O_2_).

Previous studies showed that Cst6p, Aca1p, and their paralog Sko1p have the same binding sequence *in vitro*, and the activating effects of Cst6p and Aca1p on basal expression of stress-responsive genes are only observable in the *sko1*Δ background (5, 22). Thus, a competition model between the three TFs was proposed. Here, we provide *in vivo* evidence for this model by showing that Cst6p and Sko1p can bind at the same site on target promoters (Fig. 6C). Considering that other TFs, such as Yap1p and Skn7p, also bind directly to many of these genes (Fig. 6B), integrated ChIP-exo and transcriptome analyses of all the TFs involved under various stress conditions (26) would provide new insight into this complex combinatorial regulation. On the other hand, collected microarray data showed that the expression of *CST6* itself is hardly affected by environmental changes (27), which suggests that Cst6p is connected to the transcriptional regulatory network in *S. cerevisiae* mainly through its targets.

Cst6p/Rca1p was identified as a negative regulator of genes involved in hyphal growth and cell adhesion in the human pathogens *C. albicans* (13) and *C. glabrata* (14). In *C. albicans*, the targets of Rca1p identified from transcriptome analysis have a considerable overlap with the targets of Efg1p, which is a major activator of hyphal growth. Here, the functionally conserved homolog of *EFG1* in *S. cerevisiae*, *PHD1* (28), was identified as a direct target of Cst6p. This provides a hint that Cst6p/Rca1p may mediate the suppression of hyphal growth by low levels of CO_2_ (13, 29) through Phd1p/Efg1p, which could be tested in *Candida* species. While Cst6p appears to activate the expression of *PHD1* (Fig. 3), both Cst6p and Phd1p may act as repressors in other yeasts or under specific conditions (6).

Here, we demonstrated that ChIP-exo can be used as an efficient method for mapping the precise locations where TFs bind to the genome. The present work offers three lessons about the understanding of transcriptional regulatory network: (i) it is important to use functionally active conditions to map the binding sites of TFs, (ii) genome-wide mapping of targets can reveal novel functions of TFs and suggest additional conditions for target mapping, and (iii) the transcriptional regulatory network may be far more complex than previously imagined due to the extensive combinatorial regulation and existence of TF cascades. Integrative analysis of data from different types of study is therefore critically needed in order to reconstruct complete transcriptional regulatory networks.

## MATERIALS AND METHODS

### Strains.

All the *S. cerevisiae* strains used in this study (Table 1) are derived from the uridine auxotrophic strain CEN.PK 113-5D (30), provided by P. Kötter (Frankfurt, Germany). The *cst6*Δ strain was constructed by transforming CEN.PK 113-5D with a *Kluyveromyces lactis* URA3 (*KlURA3*) marker gene (from vector pWJ1042 [31]) flanked by 45-bp upstream and downstream sequences of the *CST6* coding region and screening on synthetic complete medium lacking uracil (SC−Ura; Formedium). Similarly, the prototrophic wild-type strain was constructed by integrating *KlURA3* into the *ura3-52* locus of CEN.PK 113-5D by homologous recombination. The tagged strain *CST6*-TAP was constructed by integrating a tagging cassette containing the tandem affinity purification (TAP) tag CBP-ProtA coding sequence and *KlURA3* (as described in reference 32) into the *CST6* locus, allowing the tag to be fused in-frame to the C-terminal end of Cst6p, connected by a six glycine linker (G6). For constitutive expression of *NCE103* in the *CST6* deletion background, *CST6* in CEN.PK 113-5D was first replaced by a 159-bp fragment downstream from *CST6*, followed by *KlURA3* to obtain strain *cst6*ΔU, and then the *KlURA3* marker gene in *cst6*ΔU was looped out by the homologous recombination of the two direct-repeat 159-bp fragments on 5-fluoroorotic acid medium to obtain strain *cst6*ΔL. Plasmid pRS416-*NCE103*, constructed by inserting the *NCE103* gene flanked by the *TEF1* promoter and its own terminator between the XbaI and KpnI sites of a centromere plasmid, pRS416 (33), was transformed into *cst6*ΔL to get the *cst6*Δ(*NCE103*) strain. All the primers for cassette constructions and PCR identifications are listed in Table S5 in the supplemental material.

**TABLE 1:** Strains used in this study

Strain or genotype	Genotype or description
CEN.PK 113-5D	*MAT***a** *SUC2 MAL2-8^C^ ura3-52*
*cst6*Δ	CEN.PK 113-5D *cst6*::*KlURA3*
Wild type	CEN.PK 113-5D *ura3-52*::*KlURA3*
*CST6*-TAP	CEN.PK 113-5D *CST6*-G6-*TAP*::*KlURA3*
*cst6Δ*(*NCE103*)	CEN.PK 113-5D *cst6*Δ pRS416-*P_TEF1_*-*NCE103*

### Media and cultivations.

Single colonies from fresh agar plates were inoculated into 3 ml YPD medium or minimal medium (as described below) with 2% (wt/vol) glucose in tubes and grown for 12 to 24 h. Cells were harvested by centrifugation and resuspended in sterile water to obtain precultures. For growth in liquid medium (total volume of 20 ml in 100-ml shake flasks), strains were cultivated on a rotary shaker at 200 rpm and 30°C in minimal medium containing (liter^−1^) 7.5 g (NH_4_)_2_SO_4_, 14.4 g KH_2_PO_4_, 0.5 g MgSO_4_·7H_2_O, vitamins and trace metals as used in reference 34, and carbon sources as indicated above. For nutrient supplementation, a 100× stock containing 50 mM palmitic acid and 50 mM stearic acid dissolved in 1:1 (vol/vol) ethanol-Tween 20 was added to get a final concentration of 0.5 mM of each fatty acid, and uracil and l-arginine were added to a final concentration of 20 mg liter^−1^ of each compound. For growth assays on agar plates, cell suspensions were adjusted to an optical density at 600 nm (OD_600_) of 1.0 and diluted four times in 10-fold series. Three microliters of each dilution was spotted onto SC−Ura plates (containing yeast nitrogen base and complete supplement mixture without uracil; Formedium) supplemented with carbon sources as indicated above, and photographs were taken after 2 to 3 days of growth at 30°C.

### ChIP-exo.

Cells were cultivated in shake flasks in minimal medium with 2% (wt/vol) glucose or 1% (vol/vol) ethanol as the sole carbon source to an OD_600_ of 1.5 to 1.8 (mid-log phase). Formaldehyde with a final concentration of 1% (wt/vol) and distilled water were added to the cultures to cross-link protein-DNA complexes with an OD_600_ of 1.0 and a total volume of 100 ml. Cross-linking was performed for 12 min at room temperature with shaking and quenched by adding 2.5 M glycine to a final concentration of 125 mM. After 5 min, cells were washed twice with 20 ml cold TBS (10 mM Tris-HCl, 150 mM NaCl, pH 7.5) and frozen with liquid nitrogen. ChIP-exo was performed according to the methods in references 35 and 36, with some modifications. Briefly, cells were disrupted with glass beads on a FastPrep 24 (MP Biomedicals) and the crude cell lysate was sonicated to shear chromatin, using a Branson digital Sonifier 250 (Branson Ultrasonics). After centrifugation, the supernatant, containing chromatin fragments, was applied to IgG Sepharose 6 fast flow beads (GE Healthcare) for immunoprecipitation at 4°C with gentle rocking overnight. NEBNext end repair module, NEBNext dA-tailing module, NEBNext quick ligation module, PreCR repair mix, lambda exonuclease, and RecJ_f_ (all from New England Biolabs) were used for on-bead end repair, incorporation of 3′-d(A) DNA tails, first adaptor ligation, nick filling, and chromatin trimming, respectively. The first adaptors contain unique 6-bp index sequences (see Table S6 in the supplemental material). To elute and reverse cross-link the bound complexes, TE buffer containing 1% SDS was added to the beads and samples were incubated overnight at 65°C. After protease K (Thermo Scientific) digestion and DNA extraction with phenol-chloroform-isoamyl alcohol (Amresco, USA), the single-strand DNAs were subjected to primer extension using phi29 DNA polymerase (New England Biolabs). The products were given d(A) tails and ligated with the second adapter using the same reagents as in on-bead reactions and then amplified by PCR for 20 to 22 cycles using Phusion high-fidelity DNA polymerase (New England Biolabs). The GeneRead size selection kit (Qiagen) was used to purify DNA before and after the second incorporation of d(A) tails, second adapter ligation, and PCR. The final DNA samples were measured by using a Qubit double-stranded DNA (dsDNA) high-sensitivity (HS) assay kit (Thermo Scientific) and 2200 TapeStation (Agilent Technologies), pooled in equimolar amounts, and sequenced on the NextSeq 500 system (2 × 75 bp, mid-output mode; Illumina). All adapters and primers used in ChIP-exo are listed in Table S6.

ChIP-exo sequencing reads were mapped to reference genome assembly R64-2-1 of *S. cerevisiae* S288C with Bowtie2 (37) using the default settings to generate Sequence Alignment/Map (SAM) files. SAM files were treated with SAMtools (38) option -q 20 to remove low-quality reads and then converted to sorted BAM files. BAM files were trimmed 70 bp from the 3′ end using trimBam (http://genome.sph.umich.edu/wiki/BamUtil:_trimBam) to increase the resolution. To identify peaks and compare biological duplicates, the program GEM (39) was used. The noise level was calculated from averaged noise throughout each replicate. Binding events were manually curated in the Integrative Genomics Viewer (40) to remove redundant events representing the same binding and false-positive events with poor peak shape or poor duplication (41). Identification of target genes was done with BEDTools (42) using the closest function with the parameters -io -iu -s or -io -id -S to find any downstream or upstream targets, respectively. Gene targets with a distance of more than 1,200 bp from the binding event were sorted out.

### Bioinformatic analyses.

The binding site motif was enriched by analyzing the 100-bp sequences upstream and downstream from binding sites using MEME-ChIP 4.10.2 (43). The putative binding sites of Sko1p on promoters were predicted on the YeTFaSCo website (18). The target genes were annotated to gene ontology terms using GO Slim Mapper from SGD (27). The overlaps between Cst6p targets and targets of other TFs were obtained using the “Rank Genes by TF” tool in YEASTRACT (44).

### Quantitative real-time PCR.

Cells were cultivated to an OD_600_ of 1.5 to 1.8 (mid-log phase) in minimal medium with 2% (wt/vol) glucose or 1% (vol/vol) ethanol as the sole carbon source, harvested by centrifugation after mixing with crushed ice, frozen in liquid nitrogen, and then stored at −80°C. A FastPrep-24 homogenizer (MP Biomedicals) was used to disrupt cells, and total RNA was isolated using the RNeasy minikit (Qiagen). cDNA was synthesized using the QuantiTect reverse transcription kit (Qiagen). For quantitative PCR (qPCR), the DyNAmo flash SYBR green qPCR kit (Thermo Scientific) was used with a reaction mixture volume of 20 µl. Previously published primers for *CST6* and *NCE103* (12) were used, while primers for other genes were designed using IDT’s PrimerQuest tool. All the primers in qPCR are listed in Table S5 in the supplemental material. The PCR was performed on the Mx3005P qPCR system (Agilent Technologies). The thermal program consisted of an initial denaturation of 15 min at 95°C, 40 cycles of 10 s at 95°C and 30 s at 60°C, and a final segment of 1 min at 95°C and 30 s at 55°C followed by a ramp up to 30 s at 95°C (for the dissociation curve). *ACT1* was used as the reference gene for gene expression level comparison using the cycle threshold (ΔΔ*C_T_*) method (45). Statistical significance tests were done with a one-tailed equal variance (homoscedastic) *t* test in Microsoft Excel 2013.

### Sequence data accession number.

The ChIP-exo data have been deposited in the Gene Expression Omnibus database under the accession number GSE76154.

## SUPPLEMENTAL MATERIAL

Figure S1  Spot assay of the growth of strains. Download Figure S1, DOCX file, 0.3 MB

Table S1  The binding events of Cst6p identified by ChIP-exo. Table S1, XLSX file, 0.01 MB

Table S2  The Cst6p-binding target genes identified by ChIP-exo. Table S2, XLSX file, 0.02 MB

Table S3  GO annotation of Cst6p target genes. Table S3, XLSX file, 0.01 MB

Table S4  Transcription factors having shared binding targets with Cst6p. Table S4, XLSX file, 0.01 MB

Table S5  Primers used for strain construction and qPCR. Table S5, DOCX file, 0.02 MB

Table S6  Adapters and primers used for ChIP-exo. Table S6, DOCX file, 0.01 MB
