# Supplementary material for: Genome-Wide Mapping of Binding Sites Reveals Multiple Biological Functions of the Transcription Factor Cst6p in Saccharomyces cerevisiae
Source: mBio. 2016 May 3;7(3):e00559-16. doi: 10.1128/mBio.00559-16 (PMC4959655; doi:10.1128/mBio.00559-16)
Supplement: Figure S1 — Spot assay of the growth of strains. Download [file mbo002162810sf1.docx]

**Fig. S1.** Spot assay of the growth of strains. The method is described in the “Experimental procedures” section. Photos were taken after two (for glucose plate) or three (for ethanol plate) days of growth at 30°C.
