# Supplementary material for: Genome-Wide Mapping of Binding Sites Reveals Multiple Biological Functions of the Transcription Factor Cst6p in Saccharomyces cerevisiae
Source: mBio. 2016 May 3;7(3):e00559-16. doi: 10.1128/mBio.00559-16 (PMC4959655; doi:10.1128/mBio.00559-16)
Supplement: Table S5 — Primers used for strain construction and qPCR. [file mbo002162810st5.docx]

## Table S5. Primers used for strain construction and qPCR.

| Primer name | Sequence (5´ to 3´) | Use |
| --- | --- | --- |
| Strain construction and identification | | |
| CST6-dF | agtaaagaaaaaagtataagcccacacctttttggtaggataatgTTCGATGATGTAGTTTCTGGTT | Amplification of deletion cassette for *cst6*∆ construction |
| CST6-R | cttatcattcttgaatgaaacaccgttgtgctcaccaaaactttgGTGATTCTGGGTAGAAGATC | Amplification of deletion cassette for *cst6*∆ construction;Amplification of tagging cassette for *CST6-TAP* construction |
| CST6-dcF | TCACCTTACACGGAACATAGTTG | Identification of *cst6*∆;Amplification of upstream arm for *cst6*∆U construction |
| dcR | AAAACCAGAAACTACATCATC | Identification of *cst6*∆ and WT |
| URA3-dF | gatgttagcagaattgtcatgcaagggctccctatctactggagaTTCGATGATGTAGTTTCTGGTT | Amplification of integration cassette for WT construction |
| URA3-R | ctctaatttgtgagtttagtatacatgcatttacttataatacagGTGATTCTGGGTAGAAGATC |  |
| URA3-dcF | ATACAGTCAAATTGCAGTACTC | Identification of WT |
| CST6-F | aaggatccagacaaccaatcattacccaattctgaaaagataaaaGGAGGTGGTGGAGGTGGATC | Amplification of tagging cassette for *CST6-TAP* construction |
| CST6-cF | GATTCAGACGTTACAGAAGTGG | Identification of *CST6-TAP* |
| cR2 | TTCCATGGATCCACCTCCAC |  |
| CST6-dUR | gttgtgctcaccaaaactttgTATCCTACCAAAAAGGTGTGG | Amplification of upstream arm for *cst6*∆U construction |
| CST6-dLF | ccacacctttttggtaggataCAAAGTTTTGGTGAGCACAAC | Amplification of repeat fragment for *cst6*∆U construction |
| CST6-dLR | aaccagaaactacatcatcgaaAATGTCTTAAAGGTAATTTATACC |  |
| CST6-dDF | gatcttctacccagaatcacCTCCAAAATTCATTTACGTGAAC | Amplification of downstream arm for *cst6*∆U construction |
| CST6-dDR | TTGGGTAATGATTGGTTGTCTGG |  |
| dURA3F | TTCGATGATGTAGTTTCTGGTT | Amplification of *KlURA3* for *cst6*∆U construction |
| dURA3R | GTGATTCTGGGTAGAAGATC |  |
| CST6-diF | GCACCGAATGAAAATACCAATG | Identification of *cst6*∆U and *cst6*∆L |
| CST6-diR | GACTAAGGGGTTGACACGAG |  |
| TEF1P-F | gactctagaCACACACCATAGCTTCAAAATGTTTC | Amplification of *TEF1* promoter for pRS416-*NCE103* construction |
| TEF1P-R2 | gaagattcggtagcgctcatTTTGTAATTAAAACTTAGATTAGATTG |  |
| NCEct-F | caatctaatctaagttttaattacaaaATGAGCGCTACCGAATCTTC | Amplification of *NCE103* CDS and terminator for pRS416-*NCE103* construction |
| NCEct-R | gactggtaccACATTTGCTGGATCACAGACC |  |
| **Quantitative real-time PCR** | | |
| CST6-qF | ACCTATGACGAATCCACCAA |  |
| CST6-qR | GCTGGCTTATTTGACCGTTA |  |
| NCE103-qF | GCCACTTTAGAGTTTGCCATT |  |
| NCE103-qR | TTTGGTAAGGCTTCCCTTTGG |  |
| ACC1-qF | CTGGTGGTCAACGTGATATG |  |
| ACC1-qR | CCGGTAGGTGGGATATAGATAA |  |
| AHP1-qF | CCTGTACTGTCAGCCATATTC |  |
| AHP1-qR | TGGTGTCCTTAACACCTAAAC |  |
| ANP1-qF | GAGAGATCCAAACGGTAATCC |  |
| ANP1-qR | AAAGTGTGAGCCTGTTCTG |  |
| COR1-qF | GCGCAGAAGTCCTAATCAA |  |
| COR1-qR | CTTGGTCCCATAATCTCTTACC |  |
| GID8-qF | CAAGGAGCGTTTCCATATCA |  |
| GID8-qR | CAGTTCTACCCGTGTAACTTC |  |
| HAP4-qF | GCATCACCATGACGAGTTAG |  |
| HAP4-qR | AGTCCTGCTCTTCCATCAA |  |
| HSP12-qF | GGATAACGCTGAAGGTCAAG |  |
| HSP12-qR | ACACGACCGGAAACATATTC |  |
| HXK1-qF | CACTGACCCAGAGACTAAGA |  |
| HXK1-qR | GCTAATTTGCCCTCCAACT |  |
| ISU1-qF | ACGACTCTACTGGCGTTATTG |  |
| ISU1-qR | GGCAAGCTCAACTCCTTAGC |  |
| PHD1-qF | GCTTTATCGAATGGAGCATCTA |  |
| PHD1-qR | CTTGTTTGGGCCTCAGTATC |  |
| PYC1-qF | GTCAAGCAAGCCAAGGATAA |  |
| PYC1-qR | GCCTTCTTCACAGCATCTAC |  |
| RMI1-qF | GTTCCAGGTGTTGATGGTAG |  |
| RMI1-qR | CCACATTCCCGTCATCTTC |  |
| ROX1-qF | CAACAACAGCAGTCAAACAC |  |
| ROX1-qR | TACAGAGGAGTTCGGAGAAG |  |
| RPS3-qF | GTCGCTCAAGCTGAATCTATG |  |
| RPS3-qR | CCTTAGCACCAGATTCCATAAC |  |
| YAP1-qF | CTGGTAGCACTGGCAATAAG |  |
| YAP1-qR | CAAACGGTGACTCGGATATG |  |
| YAP6-qF | CTTCCGAACCAGAGCATATC |  |
| YAP6-qR | AGGAGGTGCAGTAGTAACA |  |
| ACT1-qF | GCCTTCTACGTTTCCATCCA |  |
| ACT1-qR | GGCCAAATCGATTCTCAAAA |  |
