# Supplementary material for: Genome-Wide Mapping of Binding Sites Reveals Multiple Biological Functions of the Transcription Factor Cst6p in Saccharomyces cerevisiae
Source: mBio. 2016 May 3;7(3):e00559-16. doi: 10.1128/mBio.00559-16 (PMC4959655; doi:10.1128/mBio.00559-16)
Supplement: Table S6 — Adapters and primers used for ChIP-exo. [file mbo002162810st6.docx]

## Table S6. Adapters and primers used for ChIP-exo.

| Adapter^a^/primer name | Sequence^b^ (5´ to 3´) | Use |
| --- | --- | --- |
| Adpt1-9 | GATCGGAAGAGCACACGTCTGAACTCCAGTCACGATCAGATCTCGTATGCCGTCTTCTGCTTGTT (65 bp)  [PHOS]CAAGCAGAAGACGGCATACGAGATCTGATCGTGACTGGAGTTCAGACGTGTGCTCTTCCGATCT (64 bp) | First adapters |
| Adpt1-10 | GATCGGAAGAGCACACGTCTGAACTCCAGTCACTAGCTTATCTCGTATGCCGTCTTCTGCTTGTT (65 bp)  [PHOS]CAAGCAGAAGACGGCATACGAGATAAGCTAGTGACTGGAGTTCAGACGTGTGCTCTTCCGATCT (64 bp) |  |
| Adpt1-11 | GATCGGAAGAGCACACGTCTGAACTCCAGTCACGGCTACATCTCGTATGCCGTCTTCTGCTTGTT (65 bp)  [PHOS]CAAGCAGAAGACGGCATACGAGATGTAGCCGTGACTGGAGTTCAGACGTGTGCTCTTCCGATCT (64 bp) |  |
| Adpt1-12 | GATCGGAAGAGCACACGTCTGAACTCCAGTCACCTTGTAATCTCGTATGCCGTCTTCTGCTTGTT (65 bp)  [PHOS]CAAGCAGAAGACGGCATACGAGATTACAAGGTGACTGGAGTTCAGACGTGTGCTCTTCCGATCT (64 bp) |  |
| Adpt2 | AATGATACGGCGACCACCGAGATCTACACTCTTTCCCTACACGACGCTCTTCCGATCT (58 bp)  GATCGGAAGAGCGTCGTGTAGGGAAAGAGTGTAGATCTCGGTGGTCGCCGTATCATTCC (59 bp) | Second adapter (universal for all samples) |
| A1extenP | CAAGCAGAAGACGGCATACGAG(22 bp) | Primer extension for single-strand DNAs eluted from beads;Final PCR enrichment of products |
| A2P | AATGATACGGCGACCACCGAGAT (23 bp) | Final PCR enrichment of products |

^a^ Double-strand adapters were synthesized by annealing forward and reverse single-strand DNA oligos (Sigma-Aldrich) as described by Lefrançois et al. 2009.

^b^ Index sequences are underlined.

**Reference**

Lefrançois, P., Euskirchen, G. M., Auerbach, R. K., Rozowsky, J., Gibson, T., *et al.* (2009) Efficient yeast ChIP-Seq using multiplex short-read DNA sequencing. *BMC Genomics*, **10**:37.
